# A Novel Design of Autonomously Healed Concrete: Towards a Vascular Healing Network

**DOI:** 10.3390/ma10010049

**Published:** 2017-01-08

**Authors:** Pieter Minnebo, Glenn Thierens, Glenn De Valck, Kim Van Tittelboom, Nele De Belie, Danny Van Hemelrijck, Eleni Tsangouri

**Affiliations:** 1 Mechanics of Materials and Construction, Vrije Universiteit Brussel, Pleinlaan 2, B-1080 Brussel, Belgium; glenn.thierens@vub.ac.be (G.T.); glenn.de.valck@vub.ac.be (G.D.V.); danny.van.hemelrijck@vub.ac.be (D.V.H.); Eleni.tsangouri@vub.ac.be (E.T.); 2 Faculty of Engineering and Architecture, Department of Structural Engineering, Magnel Laboratory for Concrete Research, Ghent University, Technologiepark-Zwijnaarde 904, B-9052 Ghent, Belgium; Kim.vantittelboom@ugent.be (K.V.T.); nele.debelie@ugent.be (N.D.B.)

**Keywords:** self-healing concrete, vascular system, multiple healing

## Abstract

Concrete is prone to crack formation in the tensile zone, which is why steel reinforcement is introduced in these zones. However, small cracks could still arise, which give liquids and gasses access to the reinforcement causing it to corrode. Self-healing concrete repairs and seals these small (300 µm) cracks, preventing the development of corrosion. In this study, a vascular system, carrying the healing agent, is developed. It consists of tubes connected to a 3D printed distribution piece. This distribution piece has four outlets that are connected to the tubes and has one inlet, which is accessible from outside. Several materials were considered for the tubes, i.e., polymethylmethacrylate, starch, inorganic phosphate cement and alumina. Three-point-bending and four-point-bending tests proved that self-healing and multiple self-healing is possible with this developed vascular system.

## 1. Introduction

Cracks are the initial symptoms of damage on concrete. Concrete is prone to cracking due to its limited tensile resistance. These cracks can be a gateway for liquids and gasses to the steel reinforcement, causing it to corrode [1]. Traditionally, macro-cracks in concrete are manually sealed by injecting polymeric or cementitious agents into the crack void. This manual repair process requires extensive preparation before application and progressive and repetitive agent injection that is cost ineffective. Moreover, often, the repair is only done at the superficial level. In the last decade, the self-healing approach of material’s design aims to develop materials that have the ability to automatically seal and repair the cracks without human intervention [2,3]. The goal of self-healing design is to increase the lifetime of structures and to decrease their maintenance cost. Focusing on concrete technology, two healing approaches are developed: autogenous and autonomous self-healing [4].

In the case of autonomous self-healing, the recovery process is due to engineered additions. The repair agent is a polymer-based or bacterial agent embedded in protective capsules [5,6,7]. The agent’s composition is similar to the one used for manual repair: adhesive pre-polymer that polymerizes in the presence of an activator (air, water or other chemical components). Previous studies have concluded that polyurethane and epoxies are the most promising polymers. Borosilicate glass has been used as encapsulation material in early and recent studies [8,9,10]. Glass has a lot of desirable properties for self-healing such as brittle behavior, it is chemically inert with commonly used healing agents, it is water resistant and industrial production of glass carriers is already possible [11]. Researchers such as Dry [9], Li [8], Van Tittelboom et al. [6] and Tsangouri et al. [12] extensively investigated the behavior of short tubular glass capsules as carrier for the healing agent.

Embedding glass into concrete has two main drawbacks: glass induces alkali–silica reactions and may not survive in the long-term aggressive chemical environment of concrete [5]. Additionally, the tubular glass capsules may not survive the concrete mixing and compaction by vibration processes. The capsules for autonomous healing have the potential of being introduced as an extra additive to the concrete mixture. Towards industrialization, the capsules should be able to survive the concrete production process. The latter is a hot topic amongst researchers [13,14,15,16]. In any case, the encapsulation material should be designed to contribute to an elongated service-life of the concrete structure and should be able to repeatedly repair the damage. A critical amount of capsules should break to provide enough healing agent to close each crack. This critical amount will also influence the mechanical properties of the concrete element.

To overcome the drawbacks of autonomous healing with multiple short capsules, i.e., cost-ineffectiveness, difficulty to place in concrete, current inability to survive the mixing process, uncertainty of enough capsules breaking and no multiple self-healing possiblilty, the vascular system provides the answer. Vascular self-healing is inspired by the blood-vessel system. The advantages are that bigger volumes of healing agent are available and that such a system can be connected to the outside, allowing (re)filling or replacing the healing agent and so taking the lifetime of the healing agent out of the equation. The design of vascular networks was successfully introduced in polymer matrix composites and remains a hot topic of research in material science [17]. Joseph et al. [18] investigated a vascular approach for concrete by embedding borosilicate glass tubes inside concrete beams with an open curved supply coming out of the structure at one side. Sangadji approached this matter by incorporating a porous network inside the concrete. To allow the healing agent to flow through the vascular network, pumps and/or atmospheric pressure can be used [19].

In this study, the commonly used encapsulation material glass is replaced with alternative brittle materials: poly methyl methacrylate (PMMA), starch, alumina or inorganic phosphate cement (IPC). First, the performance of the new encapsulation materials is evaluated by testing small-scale concrete beams, carrying short tubular capsules, under repeatable bending load. Next, a vascular network is accomplished by adding a distribution piece on both sides of long tubular capsules, which connects the system to the outside.

For the first time in the literature, the vascular network idea with an internal distribution piece is applied to design concrete with self-healing properties and the limitations of glass capsules are overcome. This new design aims to provide repeatable repair of different cracks formed in concrete. The assessment of several cracks that interact, close and reopen simultaneously is successfully done by using an integrated monitoring system: an optical method that visualizes cracking stages (digital image correlation) is combined with the acoustic emission technique that accurately locates in space and in time the damage in concrete. Both techniques are commonly applied for fracture mechanics studies of construction materials and have shown great monitoring performance in previous autonomous healing studies [12,20,21,22].

## 2. Results and Discussion

### 2.1. Capsules’ Survivability into Concrete and Their Resistance to Breakage

#### 2.1.1. PMMA and Starch Capsules

**Figure 1:**
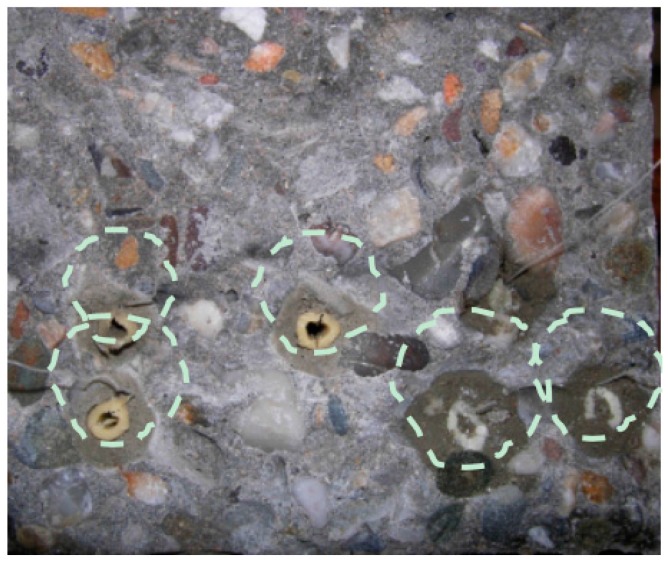
Concrete fractured surface after bending with the embedded starch-based capsules, the dotted line marks the zone surrounding the capsules.

It is found that the PMMA capsules are not brittle enough to break when cracking occurs. Additionally, after testing, the beams were cut using a diamond cutter and it was visually observed that the PU-based agent polymerized inside the capsules. Moisture was able to get inside the capsule, causing the healing agent to react. Similarly, after testing the beams carrying starch capsules, they are split in two pieces at the cracked section and the fracture surface is visually inspected. It is noted that the capsules, despite the presence of a waterproof coating, have attracted water from the concrete mixture. A circle around the capsules is observed, as shown in Figure 1. It is concluded that the coating did not protect the starch capsule and its content efficiently. Due to their chemical nature and their mechanical performance, both starch and PMMA materials are considered not suitable for this application. No further research is done in this direction.

#### 2.1.2. Cementitious and Ceramic Capsules

In contrast to starch and PMMA, the inorganic phosphate cementitious (covered by PEEK plasticizer) and the alumina ceramic capsules have shown to be compatible with the PU-based healing agent.

**Figure 2:**
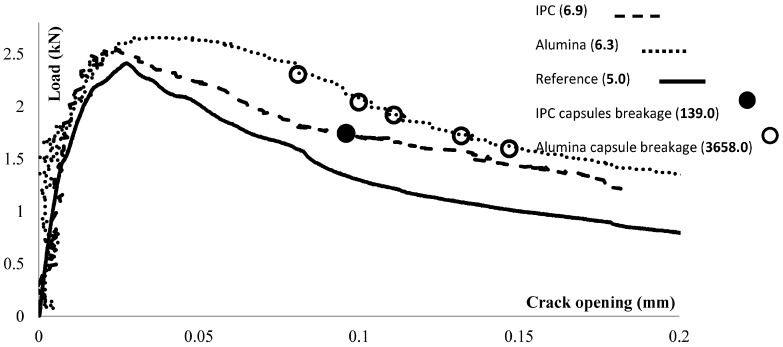
Reference beam and beams carrying ceramic (alumina) and cementitious (IPC) capsules loaded under three-point-bending.

Under three-point-bending, a macro-crack initiates from the notch and propagates along the height of the beam. The crack propagation along the capsules successfully leads to their rupture. The moment at which the capsules break is detected based on the AE hits energy analysis previously presented in [15]. In Figure 2, the loading responses of a reference beam (no capsules, black line) and of one representative beam with cementitious (striped line) and ceramic (dotted line) capsules are presented. The capsule rupture occurs instantly and emits signals with higher energy than the ones due to concrete damage. The average energy value for the concrete damage at each beam is given in bold in the figure’s label. The filled (alumina capsules) and hollow (IPC capsule) circles in this figure represent the loading stage at which high energy hits (3658 for alumina and 1396 for IPC) are emitted due to capsules rupture.

It is shown that both alumina and IPC capsules rupture only in the presence of macro-cracks wider than 80 μm. More ceramic capsules break in comparison to the cementitious ones. The fact that the capsules rupture energy is greater in the case of ceramic capsules may indicate significant resistance of alumina to damage. The excess resistance of alumina to damage can introduce local stress concentrations and interfacial detachment at the capsules’ zone and modify the concrete mechanical damage. This is not the case for the IPC capsules that break and only emit relatively low energy. The high bond strength between IPC and concrete can be the reason for the latter observation. However, few IPC capsule breakages are detected (one or two for each beam). In both cases, the agent is released in time into the crack void and the healing process is successfully activated.

### 2.2. Healing Efficiency of Ceramic and Cementitious Capsule Systems

**Figure 3:**
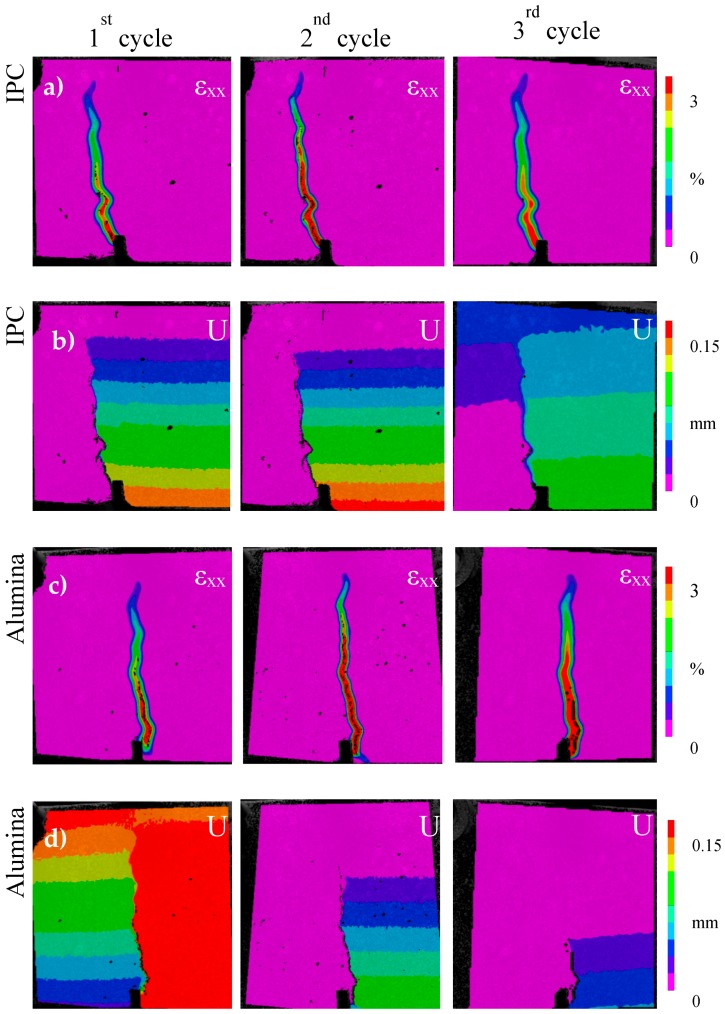
Strain (εxx) and deformation (U) maps of the beam’s side at the end of the each loading cycle: (**a**,**b**) cementitious (IPC); and (**c**,**d**) ceramic (alumina) tubes.

The efficiency is measured considering the strength and stiffness regain. In this way, the beams are reloaded up to two times after the initial damage and their mechanical response is summarized in Table 1 in which the average (two samples for each series) strength and stiffness regain is given. For IPC, higher regain of strength and stiffness is observed after healing in comparison to the reference case at which negligible resistance to damage is shown at the second loading cycle, i.e., the first reloading. It was proven that novel capsules design achieved repeatable but limited healing activation. The fact that few capsules rupture as cracking occurs can be the reason for the low strength and stiffness recovery values. As visualized by DIC strain maps captured at the end of the cycles (Figure 3a,b), the initial crack reopens after healing.

The specimen with alumina capsules achieve significant stiffness recovery but limited strength restoration. At the third loading, i.e., the second reloading, the alumina specimen show a regain in stiffness of 104%, based on Equation (3). This can be explained by a very efficient healing the second time, a lot more healing agent was able to flow into the crack. The DIC deformation (U) map at the end of the second reloading cycle shows that the crack reopens only at the bottom of the sample and does not propagate higher than the capsules level (Figure 3d). The latter observation proves that the ceramic capsules efficiently activate repeatable autonomous healing.

### 2.3. The Vascular Healing Approach: A Pipe Network

In this section, specimens with IPC or alumina long capsules, connected to a 3D printed distribution piece (i.e., a vascular system) are tested. The mechanical performance of the concrete beams after healing is evaluated for two cases:

- Three-point-bending (notched beams) simulates the guided unique cracking.
- Four-point-bending, where multiple cracking occurs, simulates more realistic loading conditions.

#### 2.3.1. Unique Cracking: The Effect of Capsules on the Mechanical Behavior

The addition of capsules in the tensile zones of the concrete specimen has a reinforcing effect. This is due to their superior tensile properties with respect to concrete. Depending on the capsule’s material, the reinforcing effect can be bigger. As a result, the ultimate strength of the beam carrying ceramic capsules is greater than there reference one and the one carrying cementitious capsules. The capsules resist cracking formation and enhance the fracture toughness, but as soon as the ultimate strength of the sample is reached, the crack propagates and the capsules instantly fail.

**Figure 4:**
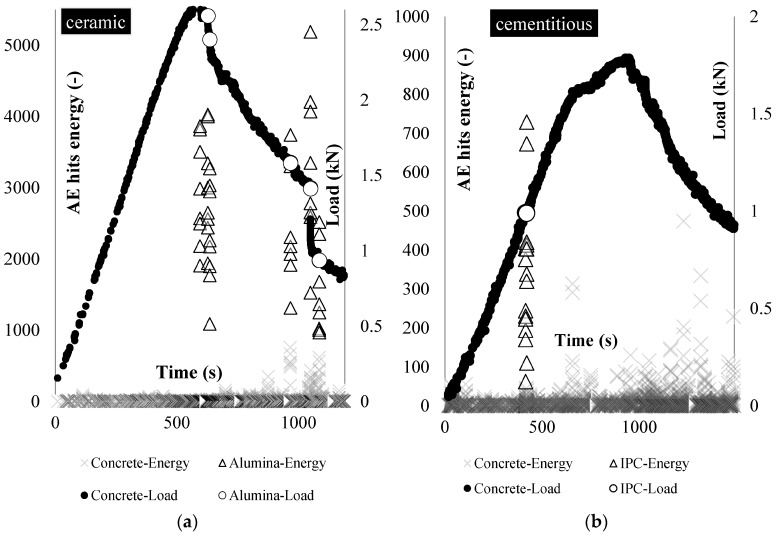
Beams carrying: (**a**) ceramic; and (**b**) cementitious piping network tested under three-point-bending: the load (black points) and the tubes rupture location (red points) based on AE energy analysis (triangular shapes).

In Figure 4, the AE hits energy (left axis and triangular points) and the flexural load (right axis and spherical points) are plotted during testing for one representative beam of each series (Figure 4a: alumina, Figure 4b: IPC). The instants at which the tubes rupture are located as high in energy AE hits [15]. The load at which the tubes rupture is indicated with circles on the loading graph.

It is shown that several alumina capsules break at the post-peak stage of loading (Figure 4a). The AE hits energy emitted at the alumina capsule rupture reached values up to 5000 (average equals to 2592). On the other hand, only two IPC capsule breakages are detected at the early loading (Figure 3b). The AE energy of the hits derived from IPC rupture reaches values up to 750 and has an average value equal to 335. 

The difference in the emitted energy between ceramic and cementitious capsules can be attributed to two parameters: firstly, the AE hits energy is relevant to the mechanical properties of the capsule material (e.g., brittleness) and the interfacial bonding developed between the concrete matrix and the capsule. Secondly, the AE energy emission depends on the fracture process that occurs locally: the local stress field is limited at the early loading stage before the crack formation and for this reason the cementitious capsule breaks “softly” emitting lower AE energy. At the ceramic capsules case, the local stress field is significant at the zone of the macro-crack that continuously widens at the post-peak stage. Therefore the capsule that resists this stress accumulates strain and as it breaks emits greater fracture energy. In any case, it is shown that the failure process of short capsules is repeated in the case of the piping system.

#### 2.3.2. Stiffness Regain after Healing of Pre-Cracked Concrete Beams

As capsules break, the healing activation process is initiated since the healing agent is released into the crack void. The concrete beams are left at their neutral, unloaded position and the agent polymerizes. 

Table 2 presents the average (two samples for each series) strength and stiffness recovery after healing for the first and second reloading cycle.

Repeatable and efficient healing can be noted for the IPC vascular system, since for the first and second reloading cycle the mechanical features are restored up to 126% and 96%, respectively.

On the contrary, the ceramic piping network achieves significant stiffness recovery but limited strength regain after healing (less than 40%). The result is in agreement with the previous findings regarding the alumina short capsules (see Table 1). The partial healing repair can be explained by partial agent delivery to the cracked surface. In other words, the alumina pipes may provide insufficient healing agent into the crack void.

**Figure 5:**
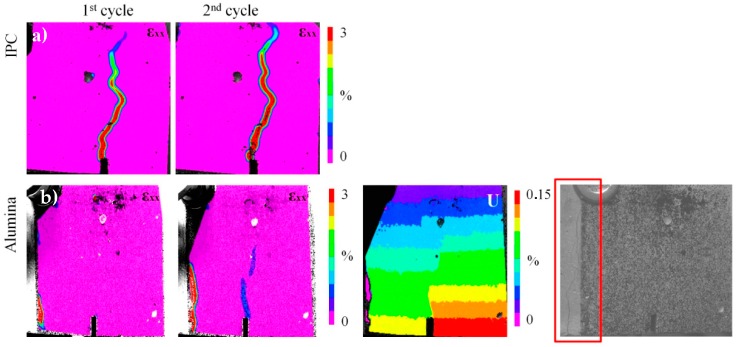
(**a**) DIC strain (ε_xx_) maps at the end of loading and reloading cycle (cementitious healing network case); and (**b**) DIC strain maps (ε_xx_) at the middle region of the beam (ceramic healing network). DIC deformation (U) map at the end of the reloading cycle. The speckle pattern is not wide enough to visualize the crack that forms at the left side of the beam. The crack is shown in the last image of this series, captured at the end of the reloading cycle.

DIC strain (ε_xx_) maps of the IPC piping network concrete beam at the end of the first and second loading cycle is presented in Figure 5a. It is shown that the initial crack reopens after healing. Once again the notch at the middle of the beam controls the crack evolution.

On the contrary, the strain (ε_xx_) maps of the ceramic piping network series show that the crack that forms at the first loading cycle is not located at the middle section of the beam (Figure 5b). The only crack formed at this initial loading cycle, is located in the region where the 3D printed distribution piece is embedded (not monitored by DIC). It is believed that the intersection between the rigid plastic distribution piece and the pipes edges is the weakest point in this testing configuration, therefore the crack initiated from this location and not from the middle notch. Unfortunately, DIC speckle pattern does not cover this region and information regarding the crack size cannot be obtained. The crack is shown only in the last image of Figure 5b presenting the side view of the beam at the end of the test. At the first reloading cycle, another crack forms at the middle zone of the sample. It is shown that the crack at the left side is efficiently repaired and, consequently, another crack forms at the next weakest point of the beam. At the end of the test, the initial crack reopens. Both cracks are visualized at the deformation (U) map taken at the end of the reloading cycle (third image in Figure 5b). The crack evolution is not monitored by DIC at the second reloading cycle, but it should be noted that the already existed cracks reopen as the 2nd reloading occurs.

#### 2.3.3. Comparison of the Capsules and Vascular Approach

**Figure 6:**
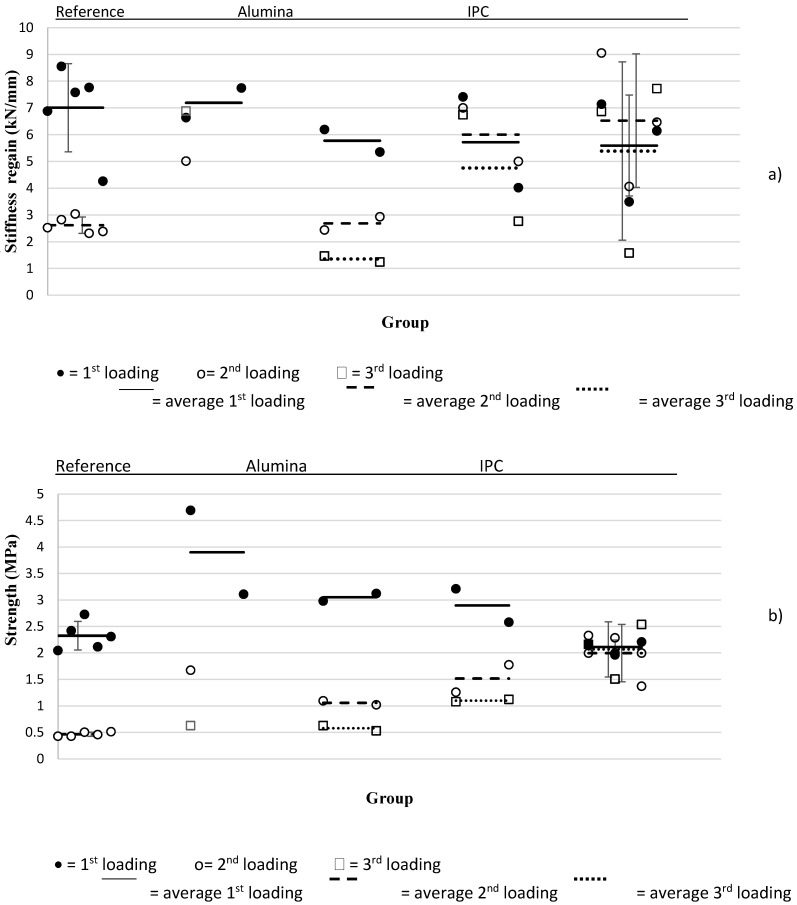
(**a**) Strength; and (**b**) stiffness values overview.

A clear difference can be seen between reloading of the capsule system and of the vascular system. The latter always shows a higher stiffness and strength recuperation (as can be seen in Figure 6a), for some even more than 100% was regained in comparison with their properties at first loading. Since the capsules contain only a limited amount of healing agent and the vascular system has a much larger supply, it is able to provide better healing efficiencies.

It can also be seen that the approach with capsules, especially with alumina capsules, has a reinforcing effect on the concrete. Once the alumina is used in the piping network the strength decreases because of the distribution piece. Regarding stiffness, this effect is not visible in the IPC case but notable in the alumina case (Figure 6b). The IPC piping network has the most constant results and only slightly alters the concrete properties in relation to the reference beam. 

Due to the small amount of specimen that survived the production process, only for the reference (*n* = 5) and the VASC IPC (*n* = 3) a statistical analysis could be performed.

### 2.4. The Piping Network Evaluation on Realistic Crack Patterns

Two series of concrete beams containing IPC and alumina (four each) piping networks were tested under four-point-bending. The test aims at evaluating the mechanical response of the embedded piping system in the presence of multiple cracks.

#### 2.4.1. The Effect of the Piping System on the Concrete Damage Processes

**Figure 7:**
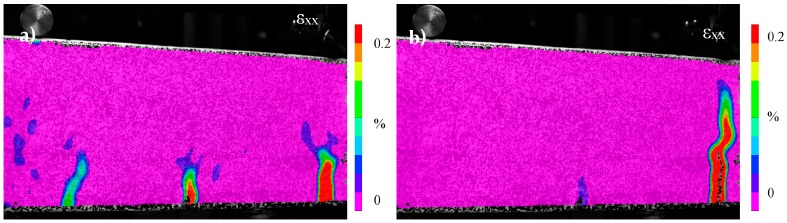
DIC strain (ε_xx_) profiles—Alumina piping system: (**a**) multiple cracks form; and (**b**) macro-crack formed at the 3D printed distribution piece.

Following the testing protocol discussed in previous paragraphs, the concrete beams are loaded up to overall deflection equal to 1 mm. DIC-strain profiles shown in Figure 7a indicate that the multiple cracking phenomena occurred as expected at the initial loading stage (before the ultimate load is reached). However, the micro-cracks shown in this loading stage do not lead to macro-cracks due to the presence of the rigid 3D printed distribution piece and the distribution of long capsules along the middle zone that guide the formation of a unique macro-crack. In other words, several micro-cracks close and only one of these cracks form finally (Figure 7b). 

**Figure 8:**
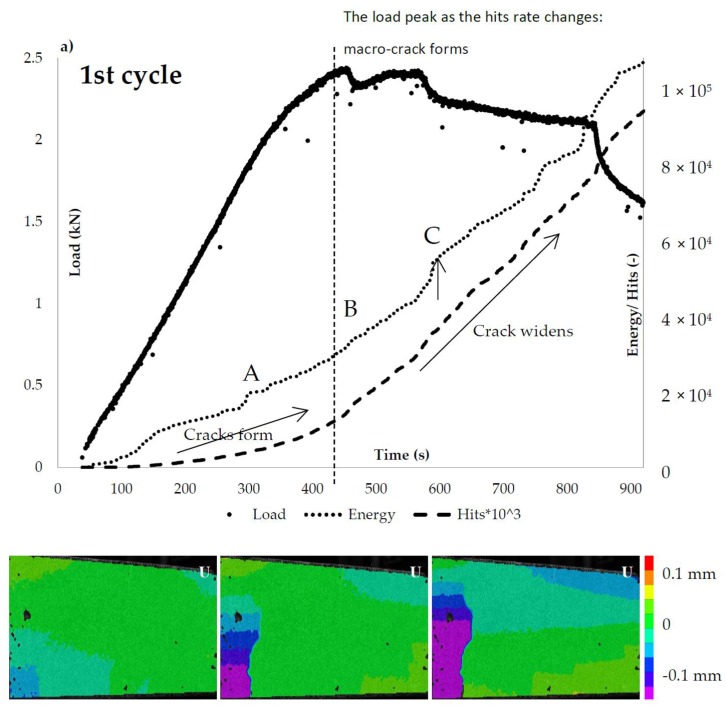

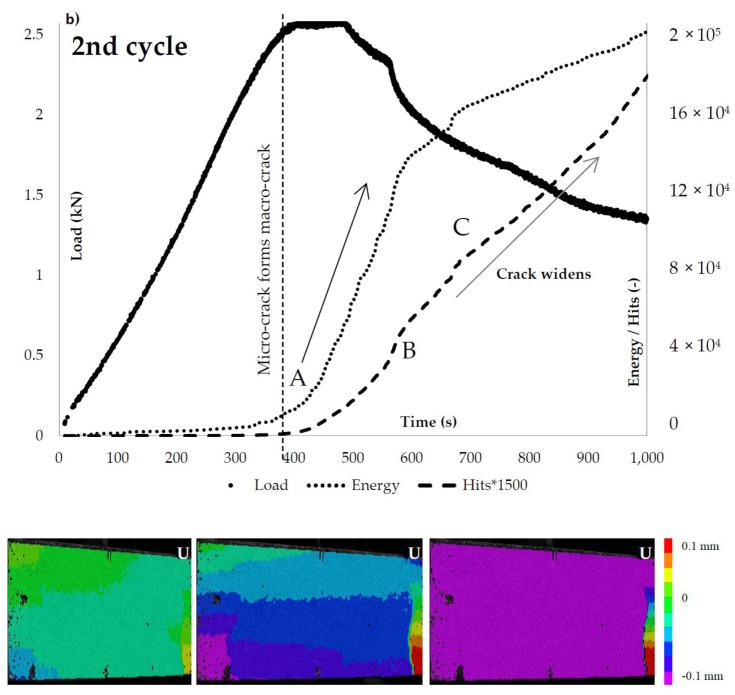
The ceramic (alumina) vascular system load response and AE hits activity: (**a**) loading (1st cycle); and (**b**) reloading (2nd cycle).

**Figure 9:**
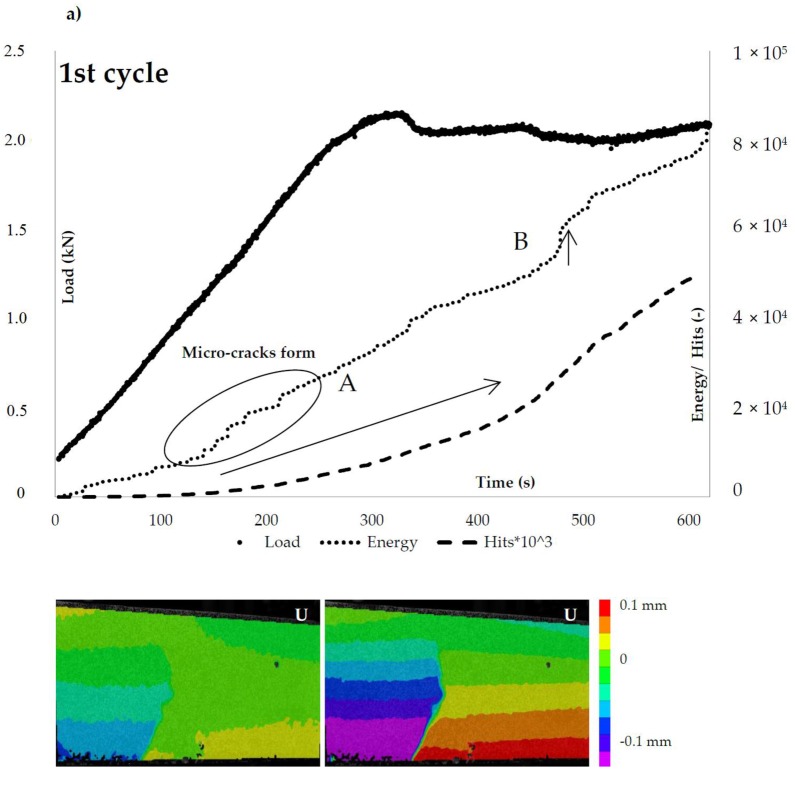

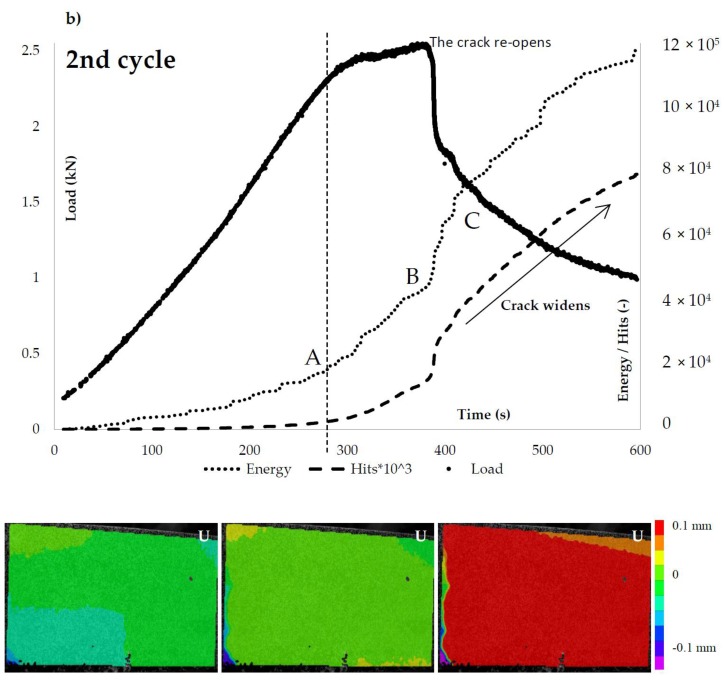
The cementitious (IPC) vascular system load response and AE hits activity: (**a**) loading (1st cycle); and (**b**) reloading (2nd cycle).

The damage propagation is monitored by DIC and AE techniques for both alumina and IPC piping systems and is presented in Figure 8 and Figure 9, respectively. To start with, in the case of alumina tubes, as the micro-cracks form and the load linearly increases the AE energy increases progressively and the hits accumulate (Figure 8a). As soon as the load linearity is lost, the macro-crack forms and the ultimate flexural load is reached. Beyond the load peak, both the cumulative energy and the AE hits rate increases as the capsules rupture and the macro-crack widens. At this post-peak stage, several load drops appear. The AE energy and hits analysis show that the load drops as soon as a capsule ruptures, e.g., point C in Figure 8. Apparently, the presence of the alumina vascular system modifies the crack morphology (a wide macro-crack instead of several ones) and the post-softening fracture processes of concrete.

This is not the case for the cementitious piping system. Once again, embedding the rigid 3D printed distribution piece controls the crack distribution leading to a unique macro-crack shown at the DIC deformation (U) maps in Figure 9a. The difference is due to the fact that the hits rate does not vary depending on the capsules rupture or the macro-damage evolution. The hits rate remains negligible till the maximum load is reached. As the ultimate load is reached, steep increase of both energy and hits rate value is obtained.

#### 2.4.2. The Healing Performance of the Vascular Systems

**Figure 10:**
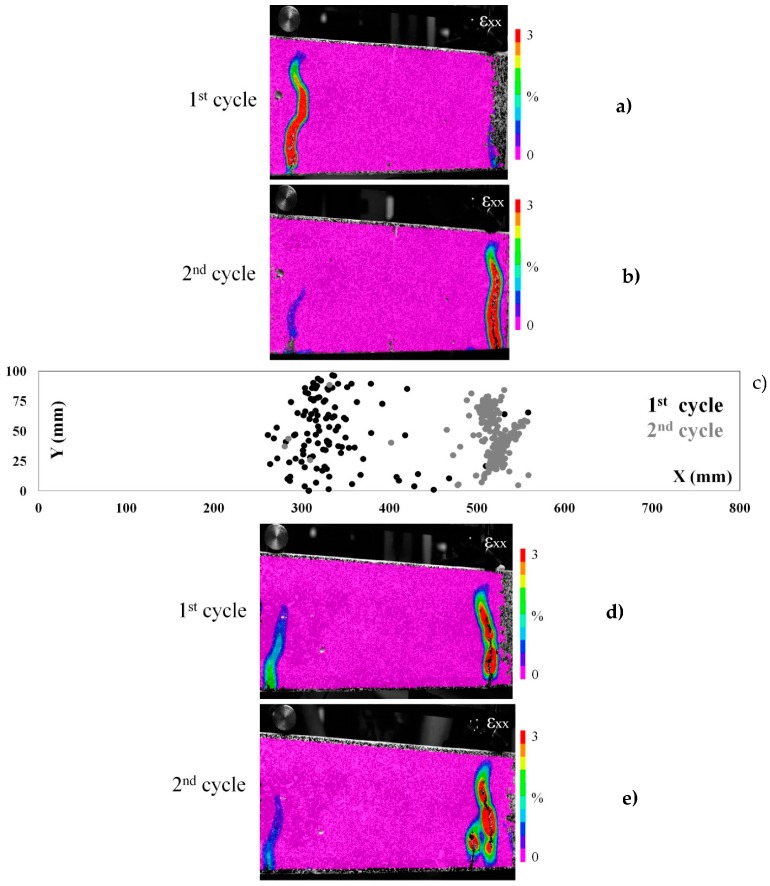
(**a**,**b**) The strain (ε_xx_) maps of beam carrying ceramic (alumina) network healing system at loading and reloading test cycles; (**c**) the AE events location map (X versus Y coordinates) is given for the first beam; and (**d**,**e**) the strain (ε_xx_) maps of a second beam with the same healing system.

**Figure 11:**
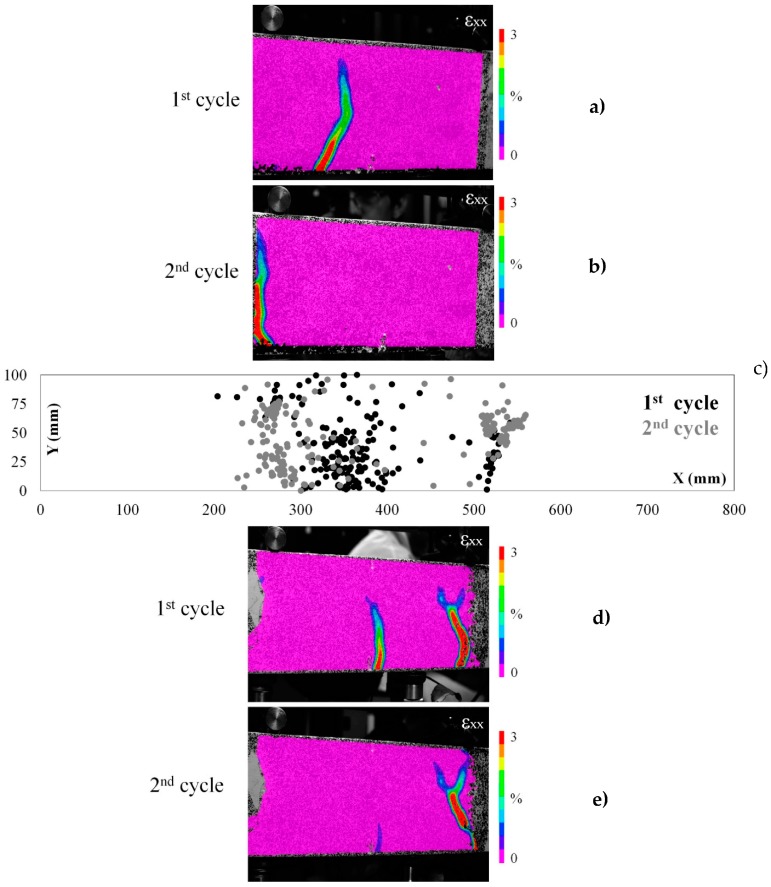
(**a**,**b**) The strain (ε_xx_) maps of beam carrying cementitious (IPC) network healing system at loading and reloading test cycles; (**c**) the AE events location map (X versus Y coordinates) is given for the first beam; and (**d**,**e**) the strain (ε_xx_) maps of a second beam with the same healing system.

The AE hits and energy remain low for both cementitious and ceramic vascular systems as the load linearly increases at the beginning of the reloading stage (Figure 8b and Figure 9b). When the peak load peak is reached, a macro-crack forms and the AE activity steeply increases. DIC strain maps show that in both cases another crack forms at the reloading cycle (Figure 10a,b,d,e and Figure 11a,b,d,e). Apparently, another crack develops since the previous crack has been successfully healed. This is great evidence that the piping system can achieve full repair of concrete damage. At any subsequent loading, the damage forms at the next weak point of the sample.

The AE location analysis confirms the DIC findings. In the graphs of X-Y coordinates (Figure 10c and Figure 11c), the events due to concrete cracking at the loading (1st cycle) and reloading (2nd cycle) are marked black and grey respectively. It can be seen that the location of damage corresponds with the strain maps of DIC. The events at the reloading cycle occur at another location and there is almost no activity at the previously damaged zone. AE shows that an additional macro-crack has been formed at the sample (Figure 11c right side) but out of the middle span zone, so this cannot be visualized by DIC and is not considered in this analysis. The latter crack should be attributed to the stress concentration developed due to the 3D printed piece intersection. Table 3 shows a strength and stiffness recovery, significantly higher than the reference.

**Figure 12:**
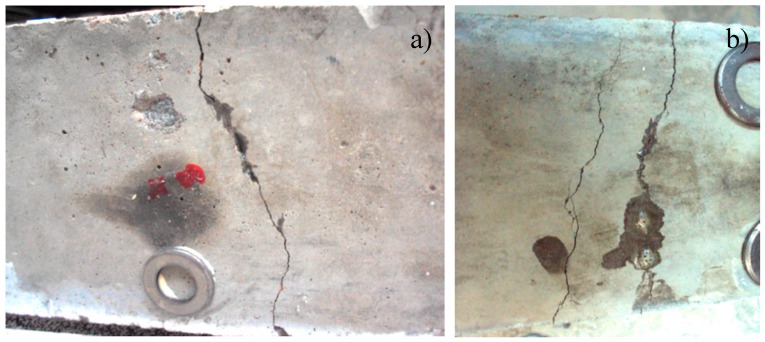
The middle of the bottom of the sample with: (**a**) cementitious; and (**b**) ceramic piping network. The healing agent leakage is visible.

In Figure 12, a bottom view from the beams is added to show that some healing agent has reached the bottom of the crack. At the second figure, two parallel cracks can be seen, with healing agent clearly visible in one of the two cracks. The crack at the left side is successfully healed and the crack at the right sides is developed at the subsequent loading cycle.

## 3. Materials and Methods

### 3.1. Concrete Beam Design

Concrete beams are designed to be tested under four-point-bending. Normal strength concrete beams are prepared with the composition presented in Table 4. The dimensions of the concrete beams are chosen according to the Rilem FMC-50 Technical Report evaluating the fracture toughness of concrete and are equal to 100 mm (width) × 100 mm (height) × 840 mm (length) [23]. A Teflon strip of 3 mm width by 10 mm height is placed in the middle section at the bottom of the concrete’s wooden mold in order to introduce a notch at the section where the maximum bending deflection is reached. This way the crack initiation is controlled.

The healing agent (MEYCO MP355 1K) is provided by BASF. This agent is a polyurethane pre-polymer, consisting of two components with, respectively, viscosities of 600 mPa·s and 70 mPa·s at 23 °C, which start to foam in moist surroundings. The expanding foaming reaction of this healing agent may lead to an increase in volume [24].

**Figure 13:**
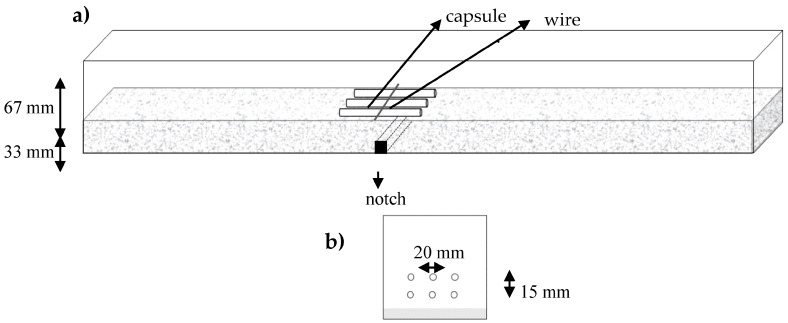
(**a**) The process of placing the first layer of three capsules inside concrete beams, the wire keeps the capsules in place; and (**b**) the cross section with two layers of capsules.

The capsules are manually filled with the agent and immediately sealed by using X60 adhesive. First a layer of concrete is put into the mold. Then three capsules are carefully placed on this concrete layer, parallel to each other and held into place by a fabric wire anchored at the mold’s sides, the distance between the capsules is around 20 mm. This process is illustrated in Figure 13.

A layer of concrete is put on top of these capsules and then a new set of three parallel capsules is put into place at a height of 45 mm from the bottom of the beam. Six capsules are used. The rest of the mold is then carefully filled with concrete.

The three-point-bending load induces stresses and forms a unique crack that initiates at the pre-crack and propagates across the beam’s height. The loading stops when the crack opening at the section above the pre-crack is equal to 300 µm considering the serviceability stress limitation (EC 2). During loading, the crack widens and the capsules standing on the crack propagation track break due to tension. The healing agent is released from the capsules and fills the crack void.

**Figure 14:**
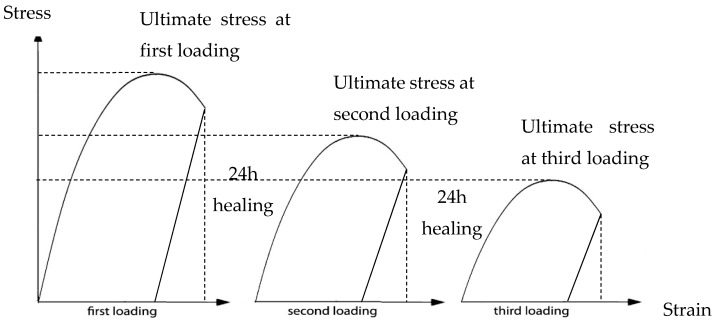
The mechanical response of the pre-cracked concrete beam under three-point-bending.

After loading until a crack width of 300 µm, the specimen is left in unloaded condition for 24 h so that the healing agent has sufficient time to polymerize. Two loading cycles, with a 24 h healing period in between, follow the initial bending and subsequently the initial crack repeatedly reopens as shown in Figure 14. The structural integrity of the beam during the second and third loading cycle provides an indication of the healing efficiency.

### 3.2. Capsules Design

Different alternatives to glass are considered for the design of the agent carriers. Their physical and mechanical properties are briefly described in the following paragraphs. The dimensions of the capsules strongly depend on the availability at the manufacturer and the production process. The presence of the capsules slightly affects the ultimate strength with respect to a normal concrete beam. The amount of healing agent in each capsule is sufficient to fill the formed crack void.

#### 3.2.1. Polymethylmethacrylate

Polymethylmethacrylate (PMMA) is a thermoplastic material that is able to resist the aggressive chemical environment of concrete, it has a density that is only half of glass and has a higher strength (see Table 5), therefore it is often used as alternative to glass, for example for windows in airplanes and headlights in cars. It is brittle, so able to break upon crack formation, and easy to manufacture. PMMA tubes were bought from the manufacturer VINK. Preliminary tests on concrete carrying the PMMA capsules should testify the effectiveness of this alternative. The series of PMMA tubes used have an outer and inner diameter of around 7 mm and 5 mm, respectively, and a length of 75 mm. Two series of notched concrete beams carried short PMMA capsules. For each sample, six capsules were filled with polyurethane agent and embedded at the middle section of the beam 20 and 35 mm (3 capsules at each level across the beam’s width) above the notch.

#### 3.2.2. Starch

**Figure 15:**
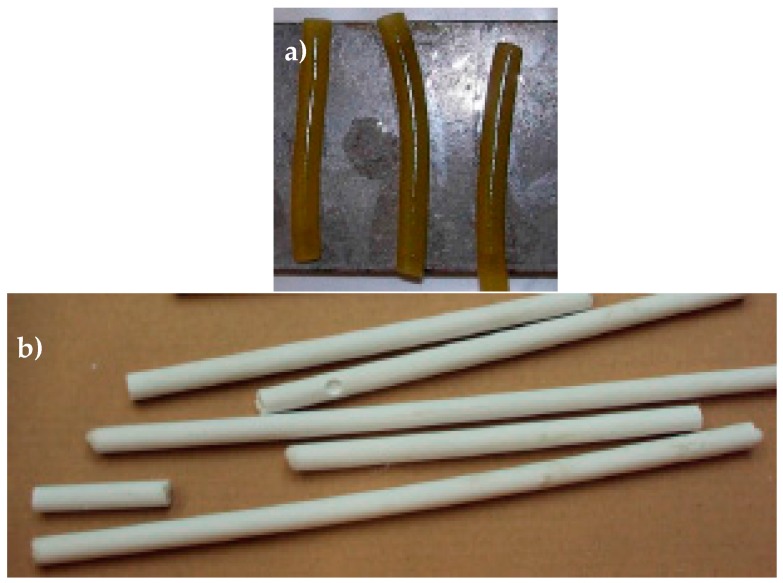
(**a**) Starch-based capsules are coated with a water-repellent spray; and (**b**) IPC tubes after curing.

Starch-based materials are brittle when dry, and flexible when exposed to humidity or increased temperatures. In a humid environment, the alpha-bonds that are encountered in starch are gradually destroyed and the material becomes flexible. Starch macaroni tubes were bought from the brand Soubry. The short (around 33 mm long) starch-based capsules used have an outer diameter of around 8 mm and an inner diameter of 2.5 mm. A water-repellent wax coating both on the inside and outside of the starch-based capsules was applied (Figure 15a) to avoid physical contact with the healing agent and softening due to the contact with the water in the concrete. Two series of notched concrete beams carried starch-based capsules. For each sample, six capsules, filled with polyurethane agent, were embedded in the middle section of the beam 20 and 35 mm (3 capsules at each level across the beam’s width) above the notch.

#### 3.2.3. Inorganic Phosphate Cement

Inorganic Phosphate Cement (IPC) is an inorganic non-alkaline ceramic material developed at the Vrije Universiteit Brussel (VUB) and has comparable properties to cement-based materials (see Table 6). It has a great compatibility with concrete and is able to resist the alkaline environment [25]. IPC consists out of a calcium silicate powder and a phosphate acid based solution of metal oxides. In order to seal the porosities a PEEK (polyether ether ketone) filler provided by DUMONT was introduced in the IPC mixture (8% per mass [26]). The tubular capsules were manufactured in a PVC mold with an outer and inner diameter equal to 7 mm and 2.5 mm, respectively (Figure 15b). The length of these capsules is approximately 50 mm.

#### 3.2.4. Alumina ceramic

Ceramics are favorable due to their brittle behavior, their great strength and stiffness (see Table 7) and great compatibility with concrete (bonding). Alumina (oxide ceramics) was provided by Ceratec. The tubes were manufactured by an extrusion process and have an outer diameter of around 3 mm and inner diameter of 2 mm. The tubes have a length of approximately 150 mm and were cut in capsules of 50 mm.

### 3.3. The Vascular Healing Network

From the design perspective, the innovation of this study is the introduction of a vascular system that can repeatedly provide sufficient healing agent at any damage location. The tubes discussed in the previous section are elongated (100 mm) and placed at the same height covering the tensile loaded section of the beam (across the beam’s length). In a first stage these beams are tested under three-point-bending and the performance of the vascular system is evaluated in the case of a unique guided crack. In a second stage, new beams with a vascular system are tested under four-point-bending simulating the realistic loading conditions. For both three-point and four-point-bending, the imposed speed was 0.04 mm/min. In the case of three-point-bending, the crack width is measured by a crack mouth opening displacement (CMOD) sensor and loading is performed until 300 µm. In the case of four-point-bending, the distance between the upper supports is 180 mm.

#### 3.3.1. The Performance of Long Tubular Capsules and the Agent Rheology

At the first step, it should be verified that the healing agent can efficiently travel through the capsule using capillary forces and potential energy caused by a height difference. It should be highlighted that the healing agent used is the one with the optimal rheological features: the viscosity of the two components of the polyurethane agent is low enough to flow through the vascular network and is high enough so that the healing agent does not flow out of the crack and is not absorbed too much by the matrix.

**Figure 16:**
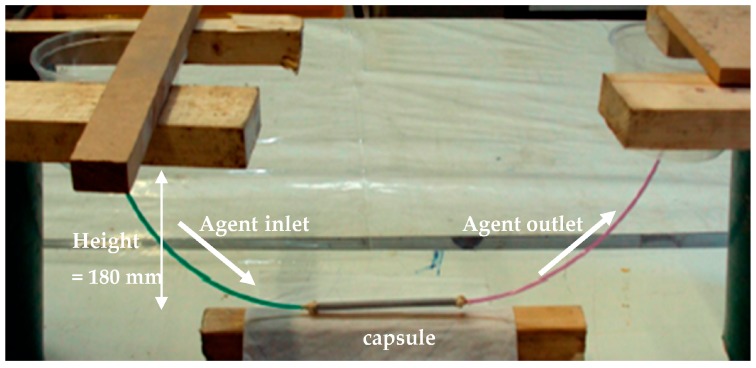
Testing the flow of PU-based healing agent in one tube.

The flowing of the polyurethane-based healing agent was tested in a simple setup as shown in Figure 16. The healing agent flows from one container, through the IPC tube of 200 mm, to the container on the other side. After travelling throughout the IPC tube, the agent successfully arrives at the agent outlet confirming the flowing ability through the capsule. Afterwards, air is pressed once again throughout the inlet in order to clean the capsule. This simple set-up confirms that by using air pressure the tube can be cleaned (i.e., no reactions took place and no agent is at the tube’s interior) after the healing process and can thus be repeatedly used.

#### 3.3.2. The Integrated Vascular Network Design

**Figure 17:**
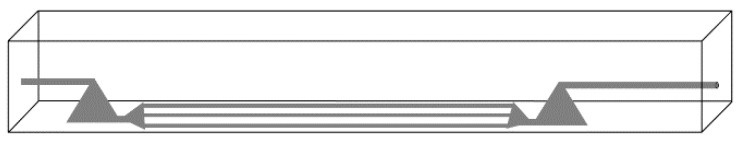
Vascular concept with tubes, accessible from the outside.

**Figure 18:**
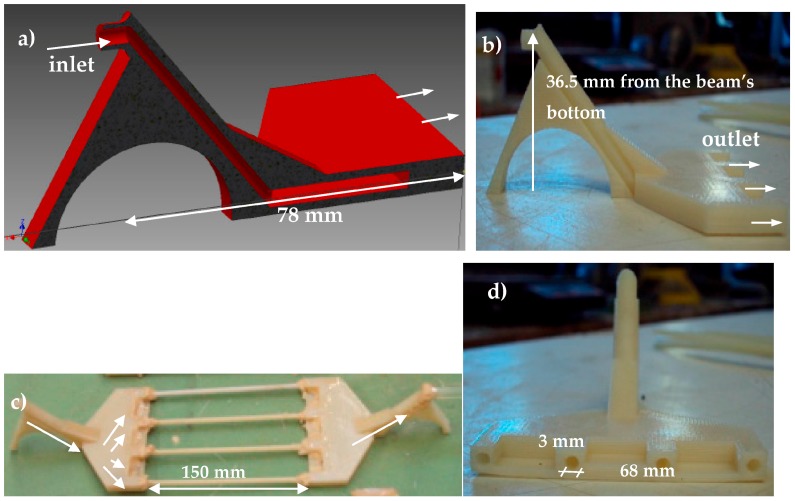
(**a**) Cross section of the 3D printed distribution piece (computer model); (**b**) side view of the printed piece; (**c**) printed pieces connected to 4 cementitious tubes; and (**d**) front view of the printed piece.

In the next step, a more complex system is designed: the inlet and outlet tubes are replaced by a distribution piece that guides the agent into the capsules, positioned at the damaged zone of the beam—note that healing is limited to the zone within the distribution pieces. This way, a reservoir system of agent is placed out of the area of damage and fills the tubes with agent only if required. This design allows repeatable self-healing capability. The concept is presented in Figure 17 and the design details of the 3D printed pieces are shown in and Figure 18. The distribution pieces are fabricated out of acrylonitrile butadiene styrene using the 3D printing technique. The specific thermoplast is chosen since it shows a good impact resistance and toughness.

A channel brings the liquid to the bottom where it is distributed over up to four tubes which can be attached to the 3D printed piece. In Figure 18a, the cross section as designed for the 3D printing fabrication is shown. In Figure 18b, the side and front view of the distribution piece are presented after the fabrication. Figure 18c shows the final fabrication of the healing system in which four long cementitious tubes are attached on a pair of distribution pieces (Figure 18d). The arrows indicate the direction of polyurethane flow throughout the system. Before placing this system in concrete, the flow of a healing agent was again successfully tested by connecting this setup to two containers, of which one is filled with healing agent (similar as was done with a single tube, described above and shown in Figure 16). The piping systems are emptied after each loading (i.e., first loading and second loading) using pressure. The system is then again filled at the subsequent reloading test. The components are assembled and then placed at the bottom of the mold. After this, concrete is carefully put inside, up to the level of the tubes. The mold is then vibrated, in order to get a good compaction of the concrete. This process is repeated two more times until the mold is completely filled with concrete.

### 3.4. Healing Performance: Mechanical Test and Monitoring Techniques

The strength and stiffness are chosen as the mechanical features that quantify the material’s cracking resistance. Both features can be accurately calculated considering the mechanical performance of a healed concrete beam that is tested before and after the healing agent is released and polymerizes into the crack void. Note that this can be different depending on the used encapsulation material. The stiffness is calculated with the following formula for the elastic (i.e., linear) region of the specimens:

(1) $$k=\frac{F}{\delta }$$

where *F* is the applied force, and *δ* is the associated displacement.

The strength is determined using the formula below:

(2) $$\sigma =\frac{3\;F_{max}L}{2bd^{2}}$$

where *F_max_* is the ultimate load, *L* is the length between the supports, *b* is the width, and *d* is the height of the specimen.

The self-healing efficiency is defined as the ratio between a property, i.e., strength or stiffness, at reloading (after healing) and at initial loading (before healing). Note that this definition includes the effect of the remaining strength/stiffness of a specimen.

(3) $$\eta_{\mathrm{property}}=\frac{Property_{\;reloading}}{Property_{initial\;loading}}$$

**Figure 19:**
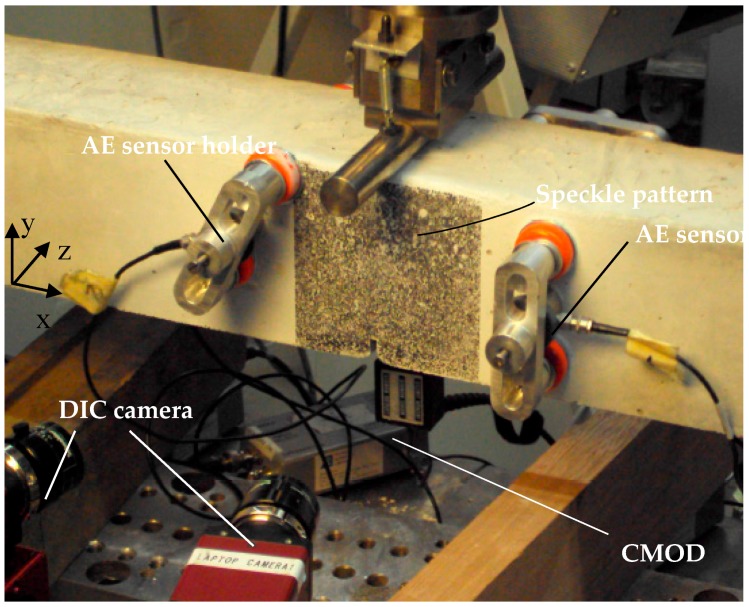
Experimental set-up for three-point-bending with AE-sensors and DIC speckle pattern.

Acoustic emission (AE) and digital image correlation (DIC) are used for inspection during the experiments. As can be seen in Figure 18, each sample has a DIC speckle pattern, manually applied by screen-printing, on the beam’s side covering the middle bending zone (100 mm × 100 mm in three-point-bending and 100 mm × 400 mm in four-point-bending). A pair of cameras forms a stereoscopic monitoring system and regularly captures high-resolution images. Given a pattern subregion, the grey intensity is measured at the reference stage and tracked at the following images as load occurs (software provided by Correlated Solutions). The full-field deformation maps (U, V, and W for X, Y, and Z coordinates defined in Figure 19) can be obtained considering the overall variation of grey values across the pattern. The respective plane strain maps (ε_xx_, ε_yy_, and ε_xy_) are obtained from the deformation derivatives.

The AE resonant sensors (resonant frequency 150 kHz) are attached by means of magnetic holders at the concrete surface at all sides of the concrete beam. The signal is received by the sensors and is amplified by 40 dB before reaching the digital storing system (8-channel AE software provided by Mistras). The received hits are filtered considering a magnitude threshold equal to 40 dB. The AE events are located in the three dimensions using the triangulation technique and considering the P-wave velocity on concrete equal to 4000 m/s. Each plastic deformation, i.e., cracking emits an elastic wave, captured by the sensors. The energy of this waveform can be quantified. Depending on the cracked material, the emitted energy is different. In the case of a heterogeneous material, such as a concrete beam with embedded capsules or vascular system, the differentiation in energy can be used to find out which material is cracking, i.e., the cementitious matrix or the capsules or tubes from the vascular system.

### 3.5. Specimen Overview and Codes

In Table 8, an overview of all the specimens, and the code appointed to each group, is given. A distinction is made between the specimens with tubes that are not connected to the outside (encapsulation system: capsule) and the specimens with a distribution piece embedded inside (encapsulation method: vascular).

## 4. Conclusions

In this study, several alternatives (ceramic, cementitious composites, starch, and PMMA) to glass material are studied to develop the optimal encapsulation system for autonomous healing applications. Both ceramic (alumina) and cementitious (IPC) were successful in this study. Additionally, inspired by vascular blood vessels in nature, a vascular network system was developed, by implementing multiple vanes (i.e., tubes) inside the beam, in order to provide repeatable autonomous healing in concrete. The polymer-based healing agent is automatically released into the concrete as soon as cracking occurs through the vascular network. The agent is delivered from a reservoir station at any location between the reservoirs into the concrete. It aims to fill and mechanically restore the damage the same way as the injection technique does traditionally on concrete repair works. It was found that the fracture toughness under bending load of concrete beams carrying ceramic tubes is significantly modified. The presence of ceramic tubes provides local strain concentration zones at the tensile section of the concrete beams and the ultimate load is increased compared to the reference case (no tubes). On the other hand, the presence of inorganic phosphate cement tubes into concrete does not affect the mechanical properties of the concrete beams and permits stable strain-softening phenomena on concrete. For this latter reason the IPC piping system appears the most viable option. In all cases, the tubes break in the presence of a macro-crack. It was proven by means of Acoustic Emission wave energy analysis that the tubes break as the crack forms and widens. The healing activation by means of tubes rupture is successfully done in all cases. At the next step, the well-studied piping system of healing agent carriers is connected with a reservoir station that stands at the beam’s vicinity and carries the polymer agent. The agent was successfully imported into the piping system through a distribution piece (accessible from outside the beam) as soon as the cracks break the capsules. The mechanical repair was observed at the second and third loading cycle proving that healing can be repeatedly achieved. Multiple self-healing phenomena are observed under four-point-bending tests at which several cracks form and propagate simultaneously. The four-point-bending test set-up on small scale concrete beams provides realistic crack patters and simulates the loading response of real-scale concrete beams under continuous load. The main contribution of this study is the design and manufacturing of the piping system by introducing innovative 3D printed agent suppliers and alternative and more efficient tubes.

## 5. Future Perspectives

In future research, the distribution piece should be adapted so that it does not cause crack formation at its position. The integrated self-healing system should be implemented in real concrete applications. In this case, the healing design should face two challenges: repair should be provided in complex micro- to macro-scale cracks and the agent should be delivered at different sections of the concrete structure. Concrete beams or slabs should be constructed and evaluated under realistic loading conditions (static and dynamic loading, continuous or instant, distributed or impact load).

Monitoring techniques should also be implemented for the self-healing concrete of the future. A sensing system that provides information on crack formation and propagation can be built using optical fibers or piezoelectric transducers. The sensing system should be embedded into the structure, have no impact on the mechanical and physical features of the concrete and be robust and accurate enough to detect cracks in order to activate in time the healing agent release from the reservoir station. The design of effective vascular healing networks and their application on real concrete structures will be the focus of our future research.

## Figures and Tables

**Table 1:** The mean mechanical recovery of the beams carrying short capsules under three-point-bending.

Sample Group	1st Reload		2nd Reload	
	η_strenght_ (%)	η_stiffness_ (%)	η_strenght_ (%)	η_stiffness_ (%)
Reference	15	9	-	-
IPC	34	46	18	23
Alumina	36	76	13	104

**Table 2:** Average mechanical recovery for the beams carrying a piping network and loaded under three-point-bending.

Sample Group	1st Reload		2nd Reload	
	η_strenght_ (%)	η_stiffness_ (%)	η_strenght_ (%)	η_stiffness_ (%)
**Reference**	15	9	-	-
**IPC**	92.3	126	99	96
**Alumina**	40	94.4	34	91

**Table 3:** The mechanical recovery for the beams carrying piping network under four-point-bending.

Sample Group	1st Reload		2nd Reload	
	η_strenght_ (%)	η_stiffness_ (%)	η_strenght_ (%)	η_stiffness_ (%)
Reference	15	9	-	-
IPC	83	101	46	108
Alumina	76	102	82	76

**Table 4:** Concrete composition.

Component	Weight Fraction (kg/m^3^)
Sand 0/4	670
Gravel 2/8	490
Gravel 8/16	790
CEM I 52.5N	300
Water	150

**Table 5:** Material properties of PMMA and Borosilicate glass.

Property	PMMA	Borosilicate Glass
Ultimate strength (MPa)	74	6.9
Tensile strain at failure (%)	0.03	0.0001
E-modulus in tension (GPa)	3.3	70
Density (kg/m^3^)	1190	2230

**Table 6:** Material properties of IPC and concrete [25].

Property	IPC	Concrete
Compressive strength (MPa)	80–120	28
Tensile strength (MPa)	6–14	2.2
E-modulus in tension (GPa)	18	30
Density (kg/m^3^)	2000	2400

**Table 7:** Material properties of alumina (as provided by Ceratec).

Property	Alumina
Bending strength (20 °C) (MPa)	220–480
E-modulus in tension (GPa)	390
Density (kg/m^3^)	3900

**Table 8:** An overview of the specimen, their code and the number of carried out loading cycles.

Encapsulation System	# of Specimens	# Loading Cycles
Reference	5	2
Tube Alumina	2	2
Tube IPC	2	2
Vascular Alumina	2	3
Vascular IPC	3	3
